# P-277. Approaching HIV MTCT Elimination: Strengthening PMTCT Strategies in the Dominican Republic

**DOI:** 10.1093/ofid/ofaf695.498

**Published:** 2026-01-11

**Authors:** Robert Paulino-Ramirez, Marisol Jimenez, Nydia Rosario, Claudia Valdez, Monica Thromann-Peynado

**Affiliations:** Universidad Iberoamericana, Santo Domingo, Distrito Nacional, Dominican Republic; Ministry of Health, Santo Domingo, Distrito Nacional, Dominican Republic; Ministry of Health, Santo Domingo, Distrito Nacional, Dominican Republic; GIS Consulting group, Santo Domingo, Distrito Nacional, Dominican Republic; Ministry of Health, Santo Domingo, Distrito Nacional, Dominican Republic

## Abstract

**Background:**

Mother-to-child transmission (MTCT) of HIV remains a public health concern in the Dominican Republic despite existing prevention strategies. Delays in diagnosis, limited ART access, and gaps in provider training hinder progress toward elimination. In response, a national intervention was implemented to integrate HIV services into maternal and child health, update clinical guidelines, and improve provider capacity.

**Methods:**

A standardized care pathway was established for pregnant women with HIV, prioritizing same-day HIV and syphilis screening, immediate ART initiation, adherence support, and structured follow-up. Providers received targeted training, and a graphical ART prescribing guide was developed. PMTCT services were expanded across 10 maternal-child hospitals, with 358 professionals trained, including doctors, nurses, counselors, lab staff, and administrators. Additionally, 14 adolescent-friendly units integrated direct HIV counseling, treatment initiation, and follow-up without referral, training 223 professionals. National pediatric HIV and perinatal care guidelines were updated. Program impact was assessed via HIV testing data from the national surveillance system, focusing on infants under two months over a 10-year comparative period.

**Results:**

Post-intervention, only 1.3% of infants born to mothers with HIV (7 of 546 tested) were HIV-positive at six weeks, reflecting a notable decline. Integration of services improved early diagnosis and ART coverage, highlighting the value of a decentralized, multisectoral model.

**Conclusion:**

A comprehensive PMTCT strategy, including early diagnosis, provider training, service expansion, and updated clinical guidelines, significantly reduced perinatal HIV transmission in the DR. These efforts have positioned the country on the verge of eliminating mother-to-child HIV transmission, representing a major public health milestone. Sustained investment and commitment to these strategies are critical to maintaining progress and securing official elimination status.

**Disclosures:**

All Authors: No reported disclosures

